# Mapping the importance of each individual element accounted by HOOS and VR-12 on 1-year patient satisfaction after primary total hip arthroplasty: a prospective institutional analysis

**DOI:** 10.1007/s00590-025-04311-7

**Published:** 2025-05-21

**Authors:** Brian Benyamini, Ahmed K. Emara, Ignacio Pasqualini, Alvaro Ibaseta, Alison K. Klika, Shujaa T. Khan, Matthew R. Zielinski, Cleveland Clinic Adult Reconstruction Research, Nicolas S. Piuzzi

**Affiliations:** 1 Cleveland Clinic Lerner College of Medicine, Cleveland, United States; 2 Cleveland Clinic, Cleveland, United States

**Keywords:** HOOS, THA, PASS, MCID

## Abstract

**Background:**

This study aimed to determine the significance of individual questions from the hip osteoarthritis outcome score (HOOS), HOOS Physical Function Shortform (PS), HOOS Joint Replacement (JR), and Veterans-Rand (VR)-12 mental composite score (MCS) in achieving a patient acceptable symptom state (PASS).

**Methods:**

A retrospective study of a prospectively collected cohort of 8236 unilateral elective primary THAs was analyzed. Responses were collected for 18 HOOS questions (pain, PS, and JR) and 6 VR-12 questions used to calculate MCS preoperatively and 1-year postoperatively. PASS was assessed through a positive response to a binary satisfaction-related question. The association between responses to questions and outcomes was examined via multivariable logistic regression models stratified by sex.

**Results:**

Sex-specific differences in PASS attainment were observed. In males, a poorer preoperative response in HOOS-PS assessing a patient’s difficulty to sit or run comfortably due to their hip was independently associated with reduced odds of achieving PASS at 1-year post-THA (odds ratio [OR] = 0.66 [95% confidence interval [CI] 0.52–0.83], *P* = 0.001, and OR = 0.83 [0.73–0.95], *P* = 0.01, respectively). Additionally, a more favorable preoperative response in the MCS metric of feeling down and blue (OR = 1.15 [95% CI 1.03–1.28], *P* = 0.01) was associated with increased PASS attainment, whereas a poorer preoperative response to having energy (OR = 0.86 [95% CI 0.76–0.97], *P* = 0.02) was associated with reduced PASS attainment. In females, only a poorer preoperative response in feeling calm and peaceful (OR = 0.87 [95% CI 0.78–0.96], *P* = 0.01) was associated with reduced odds of PASS attainment.

**Conclusion:**

Individual questions of the HOOS and VR-12 MCS were identified as being independently associated with achieving patient satisfaction at one-year following THA. Notably, predictors of satisfaction differed by sex, with both physical function and mental health factors playing a larger role in males, while mental health alone was predictive in females. Understanding specific aspects that matter most to patients, such as mental health, allows healthcare providers to tailor their care to better meet patients' needs. This approach could involve counseling, stress management techniques, and interventions aimed at reducing feelings of depression and anxiety.

**Level of evidence:**

III.

## Introduction

In the evolving landscape of orthopedic care, patient-reported outcome measures (PROMs) have become indispensable tools for assessing the success of total hip arthroplasty (THA). This shift reflects the growing emphasis on value-based healthcare, where patient satisfaction and reported outcomes are increasingly recognized as key indicators of treatment efficacy [1–3]. The Hip disability and Osteoarthritis Outcome Score (HOOS) has emerged as a prominent PROM, offering valuable insights into patients'perceptions of pain, function, and quality of life before and after THA [4]. While these standardized assessments provide a wealth of data, interpreting the raw numerical values in a clinically meaningful way remains challenging [2, 3, 5, 6].

To address the challenge of interpreting PROM scores, benchmarks such as the minimal clinically important difference (MCID) and substantial clinical benefit (SCB) have been developed [5, 7–11]. These thresholds aim to contextualize improvement values, bridging the gap between statistical significance and clinical relevance. However, achieving MCID or SCB thresholds in pain and function domains does not always correlate with patients finding their postoperative state satisfactory [11–13]. Another important measure in this context is the patient acceptable symptom state (PASS), a concept that represents the threshold beyond which patients consider themselves well [1, 7]. PASS is typically assessed through a question about whether the patient's current state is satisfactory, providing a more direct measure of patient contentment with their postoperative condition. The introduction of PASS highlights the growing recognition that patient satisfaction is a crucial outcome in orthopedic interventions. Patient satisfaction following THA is a complex construct influenced by various factors, including preoperative expectations, postoperative pain relief, functional improvement, and psychosocial elements [14, 15]. Understanding these diverse influences on satisfaction has become paramount as healthcare systems strive to optimize patient outcomes in the context of value-based care.

PROMs typically evaluate patients'functional abilities and pain experiences by assessing questions across various domains and aggregating them into a composite score [16]. This approach, while comprehensive, may obscure the relative importance of individual questions to patient satisfaction. Patients might prioritize specific aspects of their recovery differently, leading to situations where overall PROM scores improve, but patient satisfaction remains elusive [14, 17–20]. For instance, a patient might report dissatisfaction following THA due to persistent discomfort despite improvements in other areas that elevate their aggregate PROM scores. This disconnect underscores the importance of investigating the independent significance of individual PROM questions in relation to patient-reported satisfaction. By identifying which specific elements of PROMs are most closely associated with patient satisfaction, healthcare providers can better tailor their care to meet patient needs and expectations.

To address this knowledge gap, our study aims to leverage data from an extensive institutional prospective cohort to achieve two primary objectives: (1) identify key determinants associated with patient satisfaction 1 year following primary THA, and (2) determine the individual significance of questions from the HOOS, HOOS Physical Function Shortform (PS), HOOS Joint Replacement (JR), and VR-12 Mental Composite Score (MCS)—reflecting mental health—in achieving PASS [21]. By understanding the relative importance of these questions, we seek to enhance our understanding of the factors driving patient satisfaction and consequently optimize outcomes following primary THA.

## Methods

### Study design and data source

**Fig. 1:**
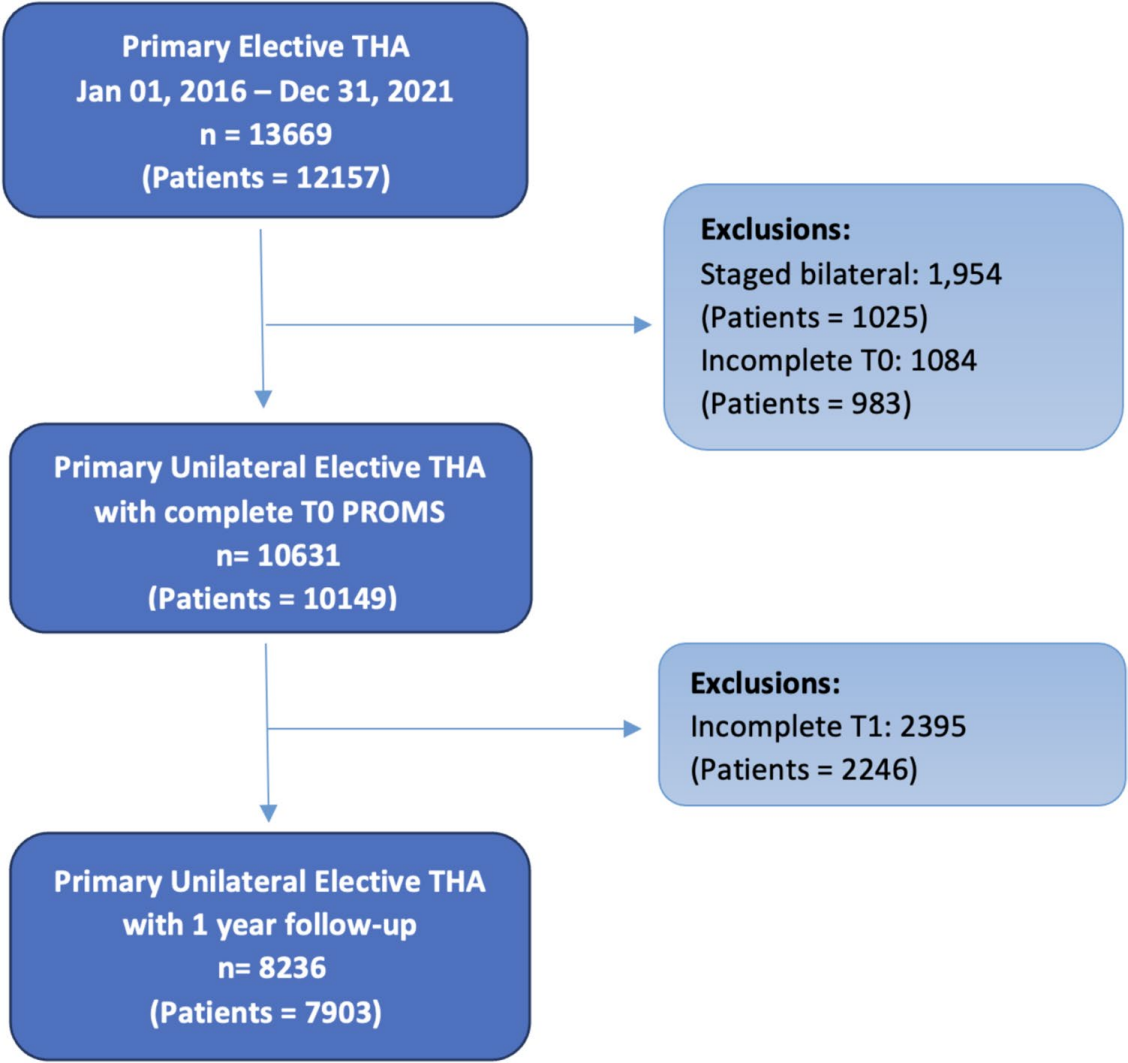
STROBE diagram illustrating the inclusion and exclusion criteria of patients in the study. THA, total hip arthroplasty; PROMs, patient-reported outcome measures; T0, baseline PROMs; T1, 1-year postoperative PROMs

A retrospective study of a prospectively collected cohort of elective primary THA patients from nine sites within a tertiary medical system, spanning January 2016 and December 2021, were screened for inclusion (*N* = 12,157 patients). Data were sourced from the OrthoMiDas (Orthopedic Minimal Data Set) Episode of Care (OME) Database, a validated database of prospectively collected general health-related PROMs and joint-specific PROMs collected preoperative and one-year postoperative [22, 23]. The OME also includes patient determinants such as age, sex, BMI, education level, race, smoking status, insurance status, as well as surgeon-reported operative details [1]. Area deprivation index (ADI) and comorbidity burden were retrieved from the electronic medical record. The study adhered to the STROBE (Strengthening the Reporting of Observational Studies in Epidemiology) statement recommendations (Fig. 1).

### Study population

Patients were deemed eligible for inclusion if they were undergoing elective unilateral primary THA due to an underlying non-oncologic etiology. Patients who were incapable of providing their own informed consent for participation in the OME cohort or expressed linguistic or physical communication barriers were excluded (Fig. 1). Furthermore, patients were excluded if preoperative PROMs were not complete. A total of 10,149 patients satisfied inclusion criteria and consented to enrollment. Of these, 7903 patients (77.9%) successfully completed 1-year follow-up PROMS, representing 8236 hips.

### Data stratification and outcomes

Baseline data on hip-specific pain and function as well as hip-related and global health-related quality of life data were collected (Table 1). Hip-specific scores were assessed using the HOOS-pain subscore (HOOS-pain) [4], HOOS-PS [24], and HOOS-JR [25] questionnaires, comprising a total of 18 questions. Overall health was evaluated using the VR-12 MCS, which includes six MCS-specific questions.

**Table 1:** Baseline patient demographics

Variable	Level	Value	*N*
Age (years)^a^		66.0 [59.0;73.0]	7903
Sex, *N* (%)	F	4576 (57.9%)	7903
	M	3327 (42.1%)	
BMI^a^		28.9 [25.4;33.3]	7901
Education^a^		14.0 [12.0;16.0]	7903
ADI^a^		51.0 [30.0;72.0]	7587
Race, *N* (%)	White	6770 (88.6%)	7637
	Other	135 (1.77%)	
	Black	732 (9.58%)	
Smoking, *N* (%)	Never	4247 (53.7%)	7903
	Quit 6 m+	2682 (33.9%)	
	Quit 0–6 m	315 (3.99%)	
	Current	659 (8.34%)	
CCI, *N* (%)	0	4366 (56.5%)	7733
	1	1450 (18.8%)	
	2	941 (12.2%)	
	3 +	976 (12.6%)	
Insurance, *N* (%)	Commercial/private/other	2642 (38.5%)	6855
	Medicaid/medicare	4115 (60.0%)	
	Self-pay	98 (1.43%)	
PASS, *N* (%)		6938 (89.2%)	7777
Baseline HOOS-Pain^a^		37.5 [25.0;47.5]	7901
Baseline HOOS-PS^a^		53.9 [38.4;62.3]	7894
Baseline HOOS-JR^a^		43.3 [32.7;53.0]	7060
Baseline MCS^a^		51.7 [41.5;60.3]	7902

Number of patients (N) used to calculate each variable are provided

ADI, Area Deprivation Index; CCI, Charlson Comorbidity Index; BMI, body mass index; PASS, Patient Acceptable Symptom State; HOOS, Hip Osteoarthritis Outcome Score; PS, HOOS physical function shortform; JR, HOOS joint replacement; MCS, Mental Composite Score

^a^Median [25 th; 75 th]

Hip-specific pain and function as well as hip-related and global health-related quality of life data (HOOS-pain, HOOS-PS, HOOS-JR, and MCS) were collected again postoperatively at 1 year (Table 2). The net improvement of aggregate score composites for these PROMs was estimated. Additionally, patients’ scaled responses to individual activity-specific questions comprising the scoring system were discretely recorded in their original 5-point scale graduated format. This provided an isolated evaluation of patients’ ability to perform specific activities from a pain and function standpoint.

**Table 2:** 1-year postoperative outcomes

Variable	Level	Value	*N*
1-Year HOOS-Pain^a^		95.0 [80.0;100]	7848
1-Year HOOS-PS^a^		95.4 [83.6;100]	7229
1-Year HOOS-JR^a^		85.3 [73.5;100]	6647
1-Year MCS^a^		56.3 [48.1;60.7]	7871
Pain difference^a^		52.5 [37.5;65.0]	7846
PS difference^a^		37.7 [25.0;50.8]	7222
JR difference^a^		41.1 [27.7;53.3]	6646
PASS threshold pain, *N* (%)	Treatment failure	2026 (25.8%)	7848
	Improved	5822 (74.2%)	
PASS threshold PS, N (%)	Treatment failure	1704 (23.6%)	7229
	Improved	5525 (76.4%)	
PASS threshold JR, N (%)	Treatment failure	2240 (33.7%)	6647
	Improved	4407 (66.3%)	

Number of patients (*N*) used to calculate each variable are provided. Differences were calculated between baseline and 1-year postoperative PROMs

HOOS, Hip Osteoarthritis Outcome Score; PS, HOOS physical function shortform; JR, HOOS joint replacement; MCS, Mental Composite Score; PASS, Patient Acceptable Symptom State

^a^Median [25 th; 75 th]

The primary outcome of this study was postoperative patient satisfaction, measured by achieving a PASS. This was determined by a positive response to the question, “Taking into account all the activity you have during your daily life, your level of pain, and also your activity limitations and participation restrictions, do you consider the current state of your hip satisfactory” [26]. Correlations between baseline PROM values and a positive response to this question were assessed to determine the relevance of individual questions in relation to patients’ postoperative satisfaction.

### Ethics approval

Approval was obtained for this study through our institution’s ethical review board.

### Statistical analysis

Continuous variables were expressed as medians and interquartile ranges (IQR). Discrete variables were depicted through counts and percentages (%). The Wilk–Shapiro test was utilized to confirm data normality. Patient responses to each activity-specific question was represented on a 5-point scale. Correlations between answers to individual PROMs questions and a positive response indicating achieving a PASS at 1 year were assessed. The association between the 5-point scaled patient responses to activity-specific questions and outcomes was examined via multivariable logistic regression models. The model was stratified by sex and adjusted for age, body mass index (BMI), education, area deprivation index (ADI), race, smoking status, and Charlson Comorbidity Index (CCI). The Gini impurity index, derived from a Random Forest approach, supplied the relative importance of variables, illustrating each variable and its contribution to the homogeneity of nodes and leaves in the resultant random forest. Variables with higher mean decreases in Gini score are interpreted as being more integral to the model, as this index is computed by assessing the average increment in node purity brought about by a split on the given variable. Variables that produce greater enhancements in node purity carry higher importance. Data handling and analysis were conducted using R software (Version 4.0; Vienna, Austria). All tests were two-sided, and used an alpha level of 0.05.

**Source of Funding** There was no funding provided for this investigation.

## Results

A total of 6938 (89.2%) patients accomplished PASS at 1 year (Table 1).

### Association between individual questions and 1 year patient satisfaction in females

Of the 18 HOOS questions that were analyzed, none were associated with PASS attainment in females (Table 3).

**Table 3:** The reported data includes patients that reported baseline and 1-year postoperative PROMs

Predictors	PASS (female)		PASS (male)	
	Odds ratio [95% CI]	*P* value	Odds ratio [95% CI]	*P* value
Age (IQR increase)	0.99 (0.85–1.15)	0.89	0.96 (0.80–1.16)	0.67
BMI (IQR increase)	1.07 (0.93–1.21)	0.34	0.92 (0.76–1.10)	0.36
Education (IQR increase)	0.90 (0.76–1.06)	0.2	1.03 (0.85–1.24)	0.76
ADI (IQR increase)	1.09 (0.90–1.32)	0.40	0.89 (0.71–1.12)	0.33
Race (other, in reference to White)	0.78 (0.34–1.76)	0.55	0.79 (0.34–1.83)	0.58
Race (Black, in reference to White)	0.72 (0.50–1.04)	0.08	0.74 (0.49–1.13)	0.17
Smoking (quit 6 m+, in reference to never)	0.89 (0.71–1.13)	0.34	0.93 (0.69–1.24)	0.60
Smoking (quit 0–6 m, in reference to never)	0.98 (0.54–1.75)	0.94	0.59 (0.34–1.02)	0.06
Smoking (current, in reference to never)	0.64 (0.44–0.94)	0.02	0.59 (0.39–0.88)	0.01
Charlson Comorbidity Index (IQR increase)	0.97 (0.91–1.05)	0.46	0.98 (0.91–1.05)	0.58
*HOOS-pain*				
1. How often do you experience hip pain?	0.86 (0.69–1.08)	0.19	1.11 (0.88–1.41)	0.38
2. What amount of hip pain have you experienced the last week while straightening your hip fully	0.99 (0.84–1.16)	0.89	0.98 (0.80–1.19)	0.82
3. What amount of hip pain have you experienced the last week while bending your hip fully	1.06 (0.90–1.25)	0.47	1.12 (0.92–1.37)	0.27
4. What amount of hip pain have you experienced the last week while walking on a flat surface	1.11 (0.91–1.36)	0.31	1.16 (0.90–1.50)	0.26
5. What amount of hip pain have you experienced the last week while going up and down the stairs	1.02 (0.86–1.22)	0.80	0.94 (0.75–1.18)	0.62
6. What amount of hip pain have you experienced the last week at night while in bed	1.03 (0.86–1.23)	0.75	0.90 (0.72–1.12)	0.35
7. What amount of hip pain have you experienced the last week while sitting or lying	0.90 (0.73–1.10)	0.29	0.98 (0.76–1.27)	0.90
8. What amount of hip pain have you experienced the last week while standing upright	0.97 (0.81–1.16)	0.71	0.87 (0.70–1.08)	0.20
9. What amount of hip pain have you experienced the last week while walking on a hard surface	0.81 (0.64–1.03)	0.08	1.02 (0.76–1.36)	0.91
10. What amount of hip pain have you experienced the last week while walking on an uneven surface	1.10 (0.88–1.37)	0.42	1.07 (0.82–1.40)	0.63
*HOOS-JR*				
11. Degree of difficulty you have experienced in the last week due to hip while rising from sitting	0.92 (0.77–1.09)	0.32	0.97 (0.78–1.21)	0.81
12. Degree of difficulty you have experienced in the last week due to hip while bending to pick up an object	1.01 (0.86–1.19)	0.90	1.11 (0.90–1.37)	0.32
13. Degree of difficulty you have experienced in the last week due to hip while lying in bed	1.10 (0.92–1.31)	0.32	1.09 (0.87–1.37)	0.46
*HOOS-PS*				
14. Degree of difficulty you have experienced in the last week due to hip while descending stairs	0.94 (0.80–1.09)	0.40	1.15 (0.93–1.42)	0.186
15. Degree of difficulty you have experienced in the last week due to hip while getting in/out of bath	1.09 (0.92–1.29)	0.31	1.04 (0.83–1.30)	0.755
16. Degree of difficulty you have experienced in the last week due to hip while sitting	0.98 (0.82–1.17)	0.84	**0.66 (0.52–0.83)**	**0.001**
17. Degree of difficulty you have experienced in the last week due to hip while running	1.00 (0.91–1.09)	0.92	**0.83 (0.73–0.95)**	**0.01**
18. Degree of difficulty you have experienced in the last week due to hip while twisting/pivoting	1.01 (0.87–1.18)	0.89	1.11 (0.92–1.34)	0.29
*MCS*				
19. During the last 4 weeks how often have you felt that you accomplished less than you wanted	0.99 (0.86–1.14)	0.88	1.08 (0.91–1.29)	0.37
20. During the last 4 weeks how often have you felt that you didn’t do work as carefully as usual	0.99 (0.86–1.14)	0.94	0.92 (0.78–1.09)	0.32
21. During the last 4 weeks how much of the time have you felt calm and peaceful	**0.87 (0.78–0.96)**	**0.01**	0.93 (0.82–1.05)	0.25
22. During the last 4 weeks how much of the time have you felt that you had a lot of energy	0.91 (0.82–1.01)	0.07	**0.86 (0.76–0.97)**	**0.02**
23. During the last 4 weeks how much of the time have you felt downhearted and blue	1.03 (0.93–1.14)	0.58	**1.15 (1.03–1.28)**	**0.01**
24. During the last 4 weeks how much of the time has your physical health or emotional problems interfered with your social activities	1.04 (0.94–1.15)	0.48	1.03 (0.90–1.16)	0.69

Data reflects the odds of successfully attaining PASS in relation to a one-point increase in preoperative HOOS question scoring

BMI, body mass index; HOOS, Hip Osteoarthritis Outcome Score; PS, HOOS physical function shortform; JR, HOOS joint replacement; MCS, Mental Composite Score; PASS, Patient Acceptable Symptom State. Bolded values indicate statistically significant findings (α < 0.05)

Of the six MCS-specific questions that were analyzed, only one was associated with PASS attainment in females (Table 2). A poorer preoperative response to feeling calm and peaceful (e.g., “some of the time”, “a little of the time”, “none of the time”) was associated with lower odds of PASS attainment (OR = 0.87 [95% CI 0.78–0.96], *P* = 0.01) (Table 2).

### Association between individual questions and 1 year patient satisfaction in males

Of the 18 HOOS questions that were analyzed, only two were independently associated with PASS attainment in males (Table 2). Poorer preoperative response to patient-reported difficulty to sit comfortably due to their hip (e.g., “mild”, “moderate”, “severe”) was independently associated with reduced odds of achieving PASS at 1-year post-THA ([OR] = 0.66 [95% CI 0.52–0.83], *P* = 0.001) (Table 2). Additionally, a poorer preoperative response to patient-reported difficulty to run comfortably due to their hip (e.g., “mild”, “moderate”, “severe”) was independently associated with reduced odds of achieving PASS at 1-year post-THA (OR = 0.83 [95% CI 0.73–0.95], *P* = 0.01). Other patient-reported activity and pain-related metrics were not significantly associated with patient satisfaction at 1-year (*P* > 0.05 each; Table 2).

Of the six MCS-specific questions that were analyzed, two were independently associated with PASS attainment in males (Table 2). A more favorable preoperative response to the patient-reported mental health parameter indicating feeling downhearted and blue (e.g., “some of the time”, “a little of the time”, “none of the time”) was independently associated with increased odds of achieving PASS at 1-year (OR = 1.15 [95% CI 1.03–1.28], *P* = 0.01) (Table 2). Furthermore, a poorer preoperative response to the energy assessment question was associated with lower odds of PASS attainment (OR = 0.86 [95% CI 0.76–0.97], *P* = 0.02) (Table 2).

### Gini index variable importance rank including baseline patient determinants

**Fig. 2:**
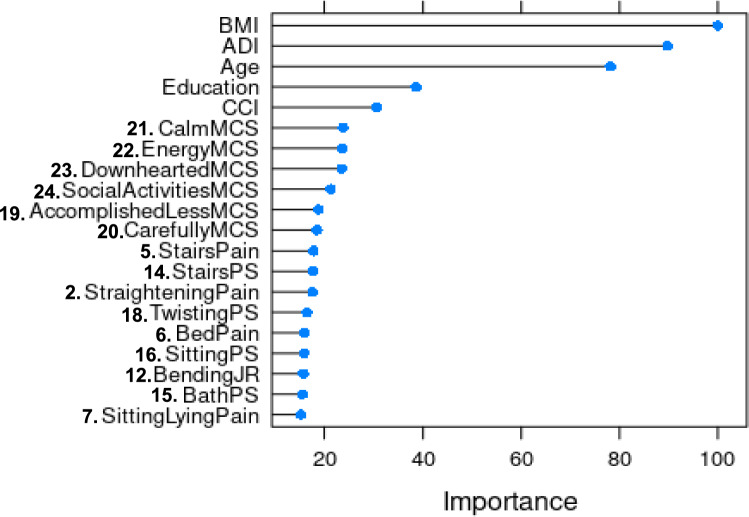
Variable importance plot for attaining PASS at 1-year post-THA stratified by baseline demographics, HOOS subscales, and MCS. Labeled numbers on the *y*-axis correlate to numbered questions in Table 3. ADI, Area Deprivation Index; CCI, Charlson Comorbidity Index; BMI, body mass index; PASS, Patient Acceptable Symptom State; HOOS, Hip Osteoarthritis Outcome Score; PS, HOOS physical function shortform; MCS, Mental Composite Score

Gini index variable importance ranking analysis showed that the top five contributors of PASS attainment at one-year postoperatively were BMI, ADI, age, education status, and CCI, respectively (Fig. 2).

## Discussion

In the evolving landscape of orthopedic care, PROMs have emerged as essential tools for assessing the success of THA. This shift reflects the growing emphasis on value-based healthcare, where patient satisfaction and reported outcomes are increasingly recognized as key indicators of treatment efficacy. PROMs typically evaluate various domains of patient experience, aggregating responses into composite scores. Yet, this approach may obscure the relative importance of individual questions to patient satisfaction. Identifying the most significant questions associated with increased patient satisfaction after THA can help identify important patient needs and expectations from the procedure. This knowledge can then be used to tailor care more effectively to meet these specific requirements, ultimately improving the overall quality of care provided. The results of this study showed that the most significant contributor of the HOOS to patient satisfaction in males was the preoperative response to the difficulty in sitting and running comfortably. Additionally, the most significant contributors of the MCS to patient satisfaction were more favorable preoperative responses in feeling downhearted and blue and having energy in males but feeling calm and peaceful in females. These findings indicate that patient satisfaction after THA is linked to the ability to perform sedentary and physically demanding activities easily, as well as enhanced psychological well-being.

Previous studies have examined the association between PROM scores and the odds of attaining patient satisfaction following THA [15, 27–31]. In a study of 207 patients, Jain et al. [32] showed that lower baseline HOOS scores predicted higher patient expectations (*P* = 0.002), which subsequently predicted higher 6 month postoperative patient satisfaction (*P* < 0.001). Furthermore, Kuo et al. [33] (*n* = 858 patients) reported that larger changes between pre- and one-year postoperative HOOS scores are associated with greater patient satisfaction. However, when other questionnaires are used, such as the Oxford Hip Score (OHS), preoperative scores are not accurate in predicting six-month postoperative satisfaction (Spearman's rank correlation coefficient = − 0.04) [34]. Other studies examined the association between preoperative mental health status and postoperative patient satisfaction. Orr et al. [35] analyzed 4034 patients and reported that patients with lower preoperative MCS scores are at increased odds of experiencing 1-year dissatisfaction (OR = 1.82, *P* < 0.001) and this association is strengthened in patients also reporting lower preoperative HOOS scores (OR = 2.07, *P* = 0.001). This finding is supported by Grits et al. [36] who showed that preoperative MCS scores greater than 60 are associated with increased patient satisfaction (OR = 2.00, *P* < 0.001). Despite the previous research performed to examine the predictive abilities of PROM scores and mental health metrics on patient satisfaction, these studies grossly analyze the data using total scores. However, no study has investigated each individual metric in the HOOS questionnaire or VR-12 MCS and their independent associations with postoperative patient satisfaction after THA.

In the present study, only two of 18 (11%) HOOS questions were independently associated with achieving patient satisfaction, and this was only observed in males. A poorer preoperative response to the question, “What degree of difficulty have you experienced in the last week due to your hip while sitting [or running]?” in males at 1 year postoperative was significantly associated with a decreased odds of being satisfied with their hip replacement. This can be explained by patients prioritizing their level of comfort while engaging in both sedentary and physically demanding activities. Sitting is a fundamental and frequent daily activity—used for working, eating, socializing, and resting—so persistent discomfort can serve as a constant reminder of their hip condition. Similarly, difficulty with running may reflect broader limitations in mobility and athleticism. For many patients, especially active individuals, regaining the ability to run may symbolize a return to high functional capacity and independence, making it a meaningful contributor to perceived surgical success.

Three of six (50%) MCS questions were independently associated with patient satisfaction. The finding in our study that half of the mental health questions were associated with postoperative satisfaction is corroborated by the high prevalence of depressive and anxiety symptoms in THA patients and their associated outcomes. For instance, Duivenvoorden et al. [37] reports that 33.6% of THA patients exhibit depressive symptoms and 27.9% experience anxiety symptoms, with many of these patients being less satisfied postoperatively compared to those with no symptoms. Mental health has a significant influence on recovery and perceived outcomes after surgery. Patients who experience improvements in mental health are more likely to report higher satisfaction levels [35, 38, 39]. This may be because positive mental health contributes to better pain management, increased motivation for rehabilitation, and a more optimistic outlook on recovery [37, 40]. Therefore, it is essential to prioritize the mental health of our patients to maximize their postoperative success in the context of THA. Integrating psychological support and interventions aimed at improving mental well-being can be beneficial. This approach could involve counseling, stress management techniques, and interventions aimed at reducing feelings of depression and anxiety.

The sex-based differences observed in our study highlight the nuanced ways in which females and males perceive and evaluate their recovery following THA. In our cohort, males demonstrated significant associations between satisfaction and both physical function (e.g., difficulty sitting and running) and mental health metrics (e.g., energy and mood). This aligns with findings from prior studies in which males exhibited higher levels of physical activity postoperatively compared to females, suggesting that regaining physical capabilities may be more strongly associated with satisfaction in male patients [41, 42]. Conversely, among females, only a single mental health item—feeling calm and peaceful—was independently associated with postoperative satisfaction. This observation is supported by literature indicating that women often report higher levels of preoperative pain and emotional distress, which can influence their postoperative perceptions of recovery and satisfaction [43, 44]. These findings underscore the importance of incorporating sex-specific considerations into preoperative counseling and postoperative care. Tailoring interventions to address mental health may be particularly beneficial for female patients, while focusing on functional recovery benchmarks may better align with male patients’ expectations and satisfaction drivers. Such an approach could enhance patient-centered outcomes and overall satisfaction after THA.

### Limitations

This study has notable limitations. Since this study relied on responses from a questionnaire, the 9.7% and 23.1% of patients that were excluded from the study for incomplete baseline and one-year postoperative PROMs, respectively, may have led to non-response bias and a subsequent reduction in generalizability of the study. Nevertheless, this study was comprised of a tertiary medical center that included 9 sites and over 27 fellowship-trained orthopedic surgeons. Therefore, the diverse settings of healthcare management and the large sample size may reduce bias and increase the generalizability of the current study.

## Conclusion

We identified individual questions of the HOOS and MCS that were independently associated with achieving patient satisfaction at one-year following total hip arthroplasty. Notably, predictors of satisfaction differed by sex, with both physical function and mental health factors playing a larger role in males, while mental health alone was predictive in females. These specific metrics that are linked with enhanced patient satisfaction after THA can help recognize patient expectations and guide the decision-making of the healthcare team. By understanding which metrics matter most, healthcare providers can tailor their care to better meet their patient needs. This approach to addressing patient priorities can optimize the delivery of care and improve overall patient satisfaction and outcomes after THA.

## Data Availability

No datasets were generated or analyzed during the current study.
